# Cryologger Ice Tracking Beacon: A Low-Cost, Open-Source Platform for Tracking Icebergs and Ice Islands

**DOI:** 10.3390/s24041044

**Published:** 2024-02-06

**Authors:** Adam Garbo, Derek Mueller

**Affiliations:** Water and Ice Research Laboratory, Department of Geography and Environmental Studies, Carleton University, 1125 Colonel By Drive, Ottawa, ON K1S5B6, Canada

**Keywords:** icebergs, ice islands, tracking beacon, Arduino, open source, hazards, Arctic, polar, low-cost, real-time data

## Abstract

Icebergs and ice islands (large, tabular icebergs) present a significant hazard to marine vessels and infrastructure at a time when demand for access to Arctic waters is increasing. There is a growing demand for in situ iceberg tracking data to monitor their drift trajectories and improve models used for operational forecasting of ice hazards, yet the high cost of commercial tracking devices often prevents monitoring at optimal spatial and temporal resolutions. Here, we provide a detailed description of the Cryologger Ice Tracking Beacon (ITB), a low-cost, robust, and user-friendly data logger and telemeter for tracking icebergs and ice islands based on the Arduino open-source electronics platform. Designed for deployments of at least 2 years with an hourly sampling interval that is remotely modifiable by the end user, the Cryologger ITB provides long-term measurements of position, temperature, pressure, pitch, roll, heading, and battery voltage. Data are transmitted via the Iridium satellite network at user-specified intervals. We present the results of field campaigns in 2018 and 2019, which saw the deployment of 16 ITBs along the coasts of Greenland and Ellesmere and Baffin islands. The overall success of these ITB deployments has demonstrated that inexpensive, open-source hardware and software can provide a reliable and cost-effective method of monitoring icebergs and ice islands in the polar regions.

## 1. Introduction

Icebergs and ice islands (large, tabular icebergs) calved from high-latitude glaciers and ice shelves pose a threat to vessels and offshore infrastructure at a time when Arctic shipping and resource exploration is increasing [1]. In the Canadian Arctic, ship traffic has increased by more than 75% over the past decade, and the continued reduction of sea ice is anticipated to further increase accessibility to the maritime Arctic [2]. Knowledge of the location of potential ice hazards is critical for ensuring safe and efficient operations [3]; however, observations from satellites, aerial reconnaissance, and shipping are often obtained on an intermittent basis and over limited areas. There is a growing demand for tracking data of representative, potentially hazardous, or unique icebergs for risk management and applied and fundamental research [4]. However, there is a lack of reliable in situ field observations of iceberg drift from these regions due in large part to the high cost of commercial tracking devices [5].

Tracking beacons have been used to measure the movements of ice in the Arctic over the past several decades [5,6,7,8,9,10,11,12,13]. Beacons could be used to complement radar and aircraft reconnaissance, providing a valuable source of information for mitigating and managing risk to industrial operations and infrastructure in ice-infested waters [14]. Data on the short-term motion of icebergs can be used to develop and enhance drift and deterioration models [15,16], while longer-term trajectories can be used to improve iceberg detection algorithms in satellite imagery [17] as well as modelling [18,19]. A better understanding of the long-term movement of ice can be used to derive drift climatologies [20], explore the influence of iceberg melt on ocean properties [21], or provide context in case studies [20,22]. In addition to positional information, ancillary data from tracking beacons, including heading and tilt, can also help determine how icebergs are drifting and deteriorating.

Current technologies used for tracking ice drift rely on Global Navigation Satellite Systems (GNSS) for positioning information, which typically provides positional accuracy of up to a few meters [23]. However, accuracy can be influenced by different sources of measurement error, such as satellite orbits, effects of the atmosphere, and receiver and antenna selection [24]. Telemetry of data is most often achieved using the Iridium satellite network [12,13,25], which offers excellent coverage over the polar regions and bi-directional data transmission in near real time. Other satellite communication platforms include ARGOS [9,26,27] and Globalstar [28,29], but these lack positional precision or do not offer coverage at high latitudes.

Some recent studies [29,30] have started to explore the use of low-cost technologies to study the drift of icebergs. The application of such technologies has the potential to greatly reduce the cost of monitoring the drift of icebergs, as reliance on expensive and proprietary commercial tracking devices presents a significant barrier to the establishment of widespread monitoring networks. For these reasons, there is a need for cost-effective instrumentation that is easily adaptable and sufficiently robust for long-term remote usage. Thus, the development of an inexpensive, user-friendly ice drift tracking beacon represents a significant advancement for the collection of in situ iceberg observations from previously data-sparse regions.

Here, we describe the design and construction of the Cryologger Ice Tracking Beacon (ITB), a low-cost, rugged tracking beacon that can be assembled at a cost that is 5–10 times less than existing commercial alternatives. We detail the initial prototype (v1.0) and a subsequent design revision (v2.0) that have been deployed on icebergs and ice islands in the Canadian Arctic. We use these 16 deployments to demonstrate the features of the ITB and highlight lessons learned as we tested these designs. Although we focus on the iceberg use case in this paper, the ITB can be used to track sea ice, glacier movement, or the position of instruments in remote environments and is equally suitable for use in the Arctic and Antarctic. With adequate waterproofing, it could also be employed as an ocean drifting buoy.

All design files, including a bill of materials, schematics and source code are made publicly available as open source under the GNU General Public License v3.0 (GPLv3) and are accessible online from a GitHub repository: https://github.com/cryologger/ice-tracking-beacon.

## 2. Materials and Methods

The Cryologger is a data logging and telemetry platform based on Arduino open-source hardware and software (www.arduino.cc). Over recent years, the Arduino has seen widespread adoption by scientists for a wide range of research applications [29,30,31,32,33,34,35]. Advances in Arduino sensor technologies continue to provide new ways to measure, monitor, and study the environment [36]. Arduino boards, shields, kits, parts, and accessories are readily available from several online retailers, such as Adafruit (www.adafruit.com) and SparkFun (www.sparkfun.com), and the circuit board designs, schematics, and related code are made completely open source.

### 2.1. Design

The design of the Cryologger platform takes into consideration several key principles, including cost-effectiveness, power efficiency, modularity, ruggedness, and ease of use. The use of low-cost, off-the-shelf components that do not require specialized tools or training to assemble makes the platform accessible to researchers, citizen scientists, and hobbyists alike. An emphasis on modularity and extensibility enables the system to be interfaced with a wide variety of easily interchangeable sensors and provides control over design complexity. The selection of components that are resistant to harsh environmental conditions also ensures continuous cold-weather operation. These design decisions, in turn, allow for the Cryologger platform to be customized for the specific research needs of the end-user, in this case as an ice tracking beacon, and suitable for deployment within a broad range of real-time environmental monitoring applications.

#### 2.1.1. Hardware

**Figure 1:**
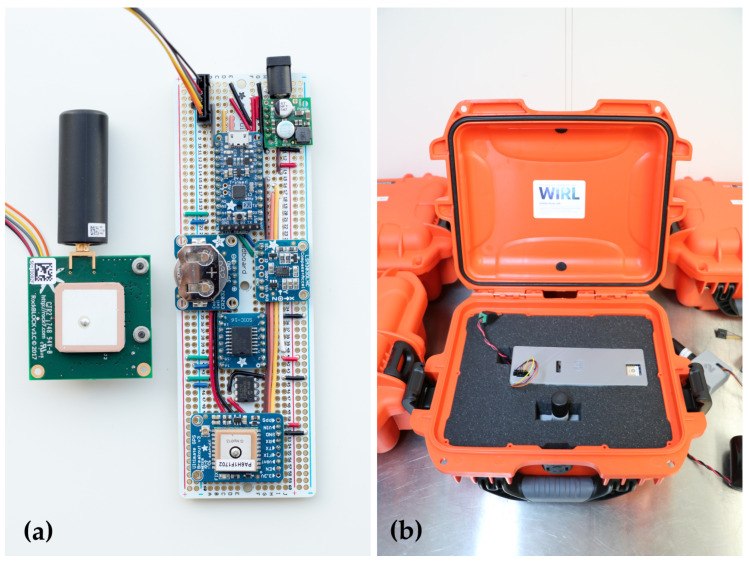
Cryologger ITB version 1.0 (**a**) Hand-assembled electronic components and RockBLOCK Iridium transceiver; (**b**) Custom 3D-printed electronics enclosure installed in Nanuk case ready for deployment.

**Figure 2:**
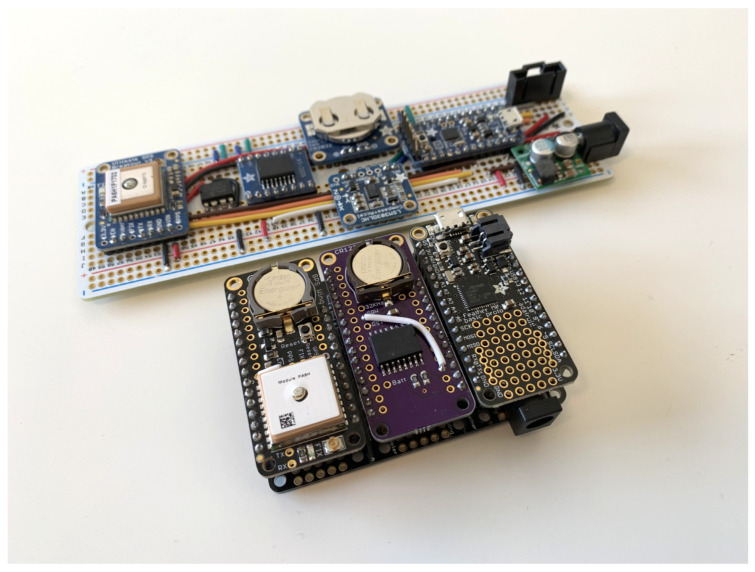
Assembled Cryologger ITB version 2.0 (foreground) based on the Adafruit Feather ecosystem of components and version 1.0 (background) for comparison. RockBLOCK Iridium transceiver not shown.

Both versions 1.0 (Figure 1) and 2.0 (Figure 2) of the Cryologger ITB were constructed primarily using Adafruit components (Table 1). A complete bill of materials is provided on the Cryologger GitHub repository. Both versions described here share the same functional design but differ in their assembly. In version 1.0, individual components were manually soldered to a protoboard, and hookup wires were used for all circuit wiring. In version 2.0, the Adafruit Feather ecosystem of components was adopted, which consists of main and daughter boards that share a common pinout configuration. This change greatly reduced the complexity of assembly by eliminating the need to measure, cut, route, and solder the many required individual hookup wires (Figure 2). The estimated assembly times for an experienced user are 4 h for version 1.0 and 2 h for version 2.0.

Version 1.0 of the Cryologger ITB was based on the Adafruit Pro Trinket microcontroller, powered by the ATmega328P processor (Microchip Technology, Chandler, AZ, USA). The software used to run the Arduino was close to the ATmega328P’s memory limit, which necessitated that the code was as memory efficient as possible. To overcome these memory constraints, the Adafruit Feather M0 microcontroller, powered by the Microchip Technology SAMD21 processor, was selected for version 2.0, which allowed for more complex code and feature-rich libraries to be used.

Both versions of the Cryologger make use of the same GNSS receiver, real-time clock (RTC), and accelerometer/magnetometer. The GNSS is based on the MTK3339 chipset (GlobalTop, Kaohsiung, Taiwan), which has a positional accuracy of 3.0 m. Power to this module can also be easily disabled when the beacon is in deep sleep. Timekeeping and alarms are achieved with the DS3231SN RTC (Analog Devices, Wilmington, DE, USA), which can maintain a clock accuracy of ±3 ppm (equivalent to ±3 s of drift over 1 year) across a wide −40 °C to 80 °C temperature range. The LSM303DLHC (STMicroelectronics, Geneva, Switzerland) accelerometer (±2 g) and magnetometer (±1.3 gauss) provide pitch, roll, and tilt-compensated heading measurements. In v1.0 of the Cryologger, an MPL3115A2 (NXP Semiconductors, Eindhoven, The Netherlands) temperature (±1 °C) and pressure (±0.1 kPa) sensor was included to measure conditions within the enclosure. In v2.0, to reduce the overall component count, the internal temperature sensor of the RTC (±3 °C) was used instead to monitor conditions inside the enclosure. Data telemetry is achieved using the RockBLOCK 9603 transceiver (Ground Control, Las Vegas, NV, USA), which is based on the Iridium 9603N module (Iridium Communications, McLean, VA, USA). An external helical antenna (M1621HCT-P-SMA; Maxtena, Germantown, MD, USA) was used in v1.0 of the beacon to achieve a better signal-to-noise ratio under adverse transmission conditions. In v2.0, it was determined that the RockBLOCK integrated patch antenna was suitable for this use case, which helped to reduce overall costs.

Power is provided to the Cryologger from a 7.4 V 38 Ah (273.6 Wh) lithium-thionyl chloride (LiSOCl2) battery pack (TLP-93121-B-AL1; Tadiran Batteries, Lake Success, NY, USA). The LiSOCl2 chemistry was selected because it is specifically engineered for operation in extremely cold conditions as low as −40 °C. This battery pack also contains hybrid-layer capacitors that absorb power spikes from the Iridium transceiver during transmissions, which prevents damage to the cells and prolongs the operational life of the pack. However, the disadvantages of this chemistry are its cost, and that it is classified as a dangerous good and must be transported according to stringent regulations [37]. Battery voltage is monitored using a simple resistive voltage divider connected to one of the microcontroller’s analog inputs. A step-down voltage regulator (Pololu Corporation, Las Vegas, NV, USA) is used to convert the voltage to 3.3/5 V to be compatible with Arduino electronics. The power consumption of the Cryologger is approximately 40 mW while idle and 130 mW during GNSS signal acquisition, and it can peak as high as 2.25 W while transmitting data via the RockBLOCK transceiver. When in deep sleep, power consumption decreases to approximately 1 mW. Once deployed, the Cryologger ITB has a maximum lifespan of up to 4 years at a 1-h sampling and 3-h transmission schedule (i.e., 35,000 measurements and 12,000 transmission cycles; see Section 3). However, the effective lifespan is dependent on several factors, including whether the RockBLOCK transceiver has an obstructed view of the sky, which can result in much longer transmission times and greater power consumption. When combined with extremely cold temperatures, further reductions in power efficiency can occur and lead to premature battery depletion.

To protect the electronics from the elements, the ITBs are housed in rugged Nanuk cases (Plasticase, Terrebonne, QC, Canada) (Figure 1b). These cases are impact resistant, have an IP67 rating against water ingress, and are rated to temperatures as low as −30 °C. In v1.0, the electronics were also placed in a 3D-printed case for extra protection, which was not possible in v2.0 due to time constraints before deployment. To improve the grip of the case on the ice surface and prevent the beacons from being blown off the iceberg by the wind, a simple solution of wooden boards with screws drilled into them was affixed to the bottom of the case with rope. The wood also provides added buoyancy if the beacon falls into the water and acts as an insulating layer between the case and the ice surface.

#### 2.1.2. Software

The Cryologger platform is programmed in C++ and is extensible, meaning that modifications to system functionality can be easily made by enabling or disabling specific blocks of code. The code is written in and uploaded using the Arduino Integrated Development Environment (IDE; v1.8.19), which benefits from numerous software libraries available from the open-source community. These libraries reduce complexity by helping to interface with components and include examples of real-world applications that can guide users who are new to programming. Two key libraries used to develop the software for the Cryologger ITB are the TinyGPS and Iridium SBD libraries, written by Mikal Hart [38,39]. These libraries enabled the functionality required to communicate with the GNSS receiver and RockBLOCK transceiver, respectively.

The programming logic optimizes sleep and wake cycles to minimize overall power consumption. When initially powered on, the system attempts to acquire a signal from the GNSS receiver and synchronizes the RTC with the current date and time. It then sets an alarm for the initial sampling interval and enters a low-power, deep-sleep mode. When the alarm triggers, the system wakes, records the time, and acquires a GNSS position. Next, measurements of all onboard sensors are collected, including temperature, pressure, battery voltage, and orientation of the beacon (i.e., pitch, roll, and tilt-compensated heading) from the accelerometer and magnetometer. These data are stored in memory, and when the software determines the appropriate transmission interval has been met, it attempts to transmit the data using the RockBLOCK satellite transceiver. The software then disables power to all components, sets the next alarm, and returns to sleep.

Several software design techniques are intentionally utilized to ensure the long-term reliability of the Cryologger ITB. First, the Watchdog Timer module (WDT) of the Arduino microcontroller is used to ensure a system reset is triggered in the case of a software malfunction that prevents normal operation. The WDT is a timer with a fixed period, which counts the ticks of its clock until it is periodically reset every 8 s, or the timeout period of 16 s is reached. In the event of a timeout, the WDT will trigger a system reset. This functionality is intended to allow the program to gracefully recover from malfunctions that could otherwise cause it to become inoperable. Additionally, the software provides redundancy in the event of a sensor or component failure. For example, if a sensor fails to initialize, future attempts to obtain a measurement with the concerned instrument will be skipped. Finally, when transmitting data via the RockBLOCK transceiver, the software checks for the presence of incoming messages sent by the end-user, which can be used to issue a command to force the system to reset manually.

#### 2.1.3. Data Transmission and Processing

The Cryologger ITB nominally records its position and sensor measurements on an hourly basis and stores them in memory until a transmission interval of 3 h is reached. Data are transmitted via the Iridium satellite network as a Short Burst Data (SBD) message, which has a maximum size of 340 bytes. Each data sample is stored as a 30-byte binary message (Table 2). This minimizes the total number of required transmissions and reduces the overall power consumption of the system by allowing more samples to be sent in a single message. Each message is attempted to be transmitted for up to 180 s, and if unsuccessful, the message is stored in a temporary buffer and reattempted at the next transmission interval. Both the sampling and transmission frequency of individual ITBs can be remotely modified by the end-user by transmitting an SBD message to its International Mobile Equipment Identity (IMEI), the unique identifier associated with each Iridium transceiver. Successfully transmitted SBD messages are received by an Iridium ground station and sent to Ground Control’s server. These data are then forwarded to Amazon Web Services (AWS), where they are decoded using a Python script, stored in a database, and visualized on a website. Raw data collected from Cryologger ITBs can be viewed in near real time and is freely available for download at https://cryologger.org. It should be noted that there is a subscription cost to send data through the Iridium network that varies by provider. For hourly data (Table 2), the cost is on the order of 35 USD a month, not including activation fees.

### 2.2. Deployments

**Figure 3:**
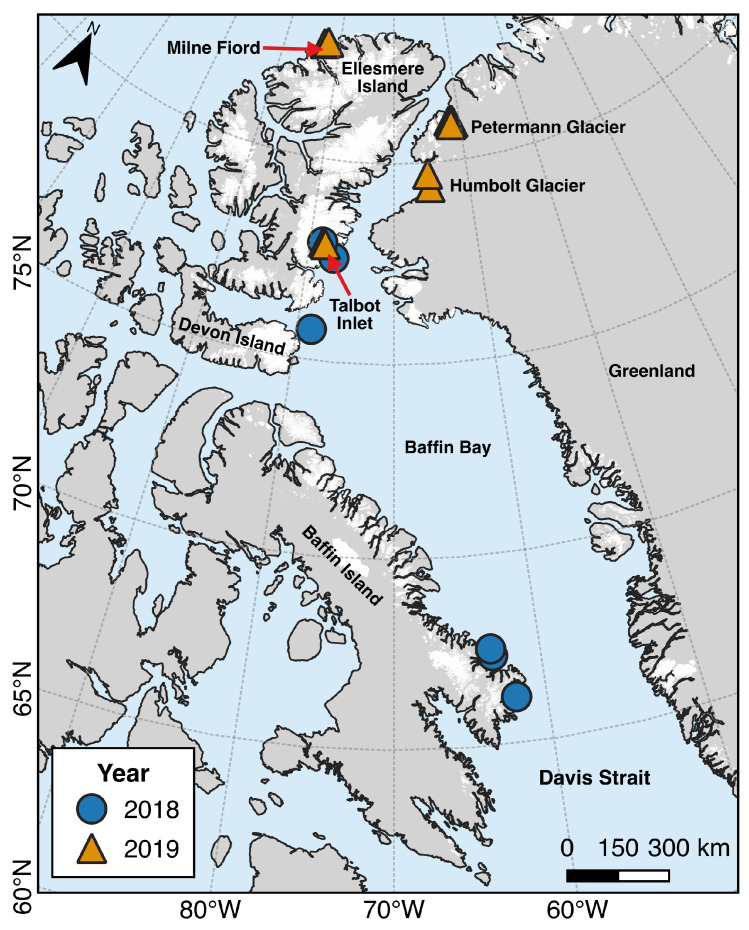
Locations and number of Cryologger ITB deployments made in the Canadian Arctic during 2018 and 2019 field campaigns. ITBs were deployed from the CCGS Amundsen in 2018 in Talbot Inlet (2) and the coastal regions of Devon Island (1) and Baffin Island (3). In 2019, ITBs were deployed in Milne Fiord (2) during a scientific field campaign. ITBs were also deployed that year from the CCGS Amundsen on Petermann Glacier (4), near Humbolt Glacier (2) and in Talbot Inlet (2).

**Figure 4:**
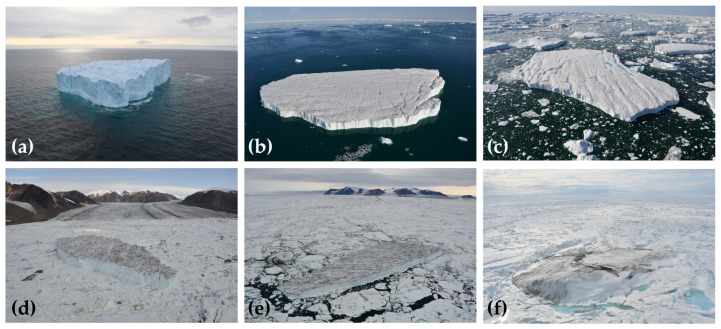
Examples of suitable iceberg targets instrumented with Cryologger ITBs during the 2018 and 2019 field seasons. Measurements of iceberg dimensions derived from satellite imagery. (**a**) 300434063415110 (270 m × 204 m); (**b**) 300434063494100 (490 m × 300 m); (**c**) 300434063392070 (600 m × 330 m); (**d**) 300434063394110 (760 m × 260 m); (**e**) 300434063495310 (700 m × 240 m); (**f**) 300234065254740 ice island fragment.

A total of 14 Cryologger ITBs were deployed during the 2018 and 2019 summer expeditions of the CCGS Amundsen icebreaker, and 2 ITBs were also deployed by helicopter in the summer of 2019 on ice island fragments located near the Milne Ice Shelf, Ellesmere Island (Figure 3). Deployments from the CCGS Amundsen were made by helicopter on icebergs and ice islands (Figure 4) along the coast of Ellesmere Island, Baffin Island, and Greenland. An additional 4 beacons were deployed on the terminus of the Petermann Glacier in anticipation of the next major calving event, which could produce an ice island up to approximately 185 km^2^ in size (determined by delineating the area down-glacier from rifts in a Sentinel-2 satellite image). The overall suitability of potential targets was determined by assessing the iceberg’s size, shape, and location. Additionally, strict safety protocols specific to our deployment context were implemented to mitigate the inherent risks associated with working on icebergs. This included only landing on sizable icebergs that are tabular in shape, which are not prone to roll, minimizing time spent on the ice surface, and maintaining a safe distance away from edges. Where possible, tracking beacons were deployed on icebergs that were large enough to potentially survive drifting south to the Grand Banks of Newfoundland Labrador and far enough away from the coast to increase the chances of being carried southward by the currents and avoid becoming grounded in shallow coastal areas typical of these areas. Detailed information regarding the timing and locations of Cryologger ITB deployments, as well as estimates of the iceberg’s physical characteristics, can be found in the Iceberg Tracking Beacon Database [40], a new joint project between the Canadian Ice Service and Carleton University that aims to compile historical iceberg tracking beacon data from the northern hemisphere into a single data repository.

**Figure 5:**
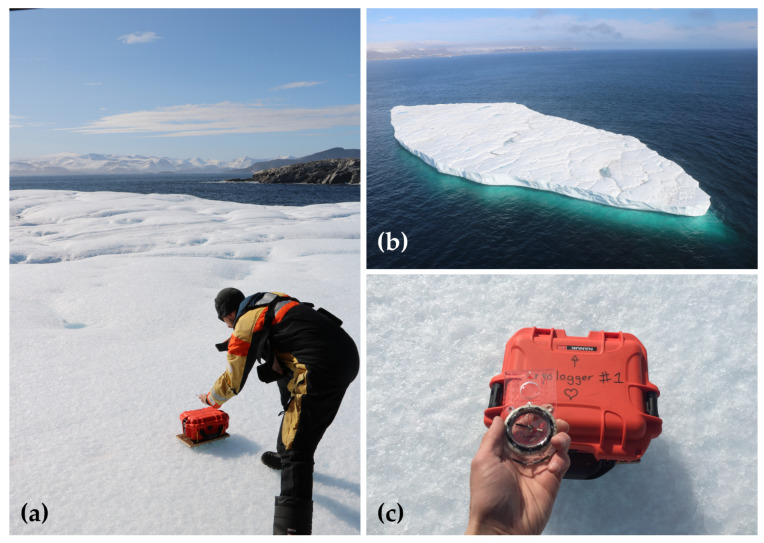
Example of a typical Cryologger ITB deployment: (**a**) Hand deployment of ITB 300434063418130 on 3 September 2018. (**b**) Aerial photo of iceberg target located on the southern coast of Baffin Island, Nunavut (approx. 600 m × 330 m); (**c**) Measurement of the magnetic compass heading of ITB at time of deployment.

At the time of deployment, information about the iceberg’s shape, size, and position was recorded (Figure 5a). Estimates of the iceberg’s waterline length were made visually from the air (Figure 5b), and the helicopter’s onboard altimeter (accuracy of ±0.9 m) was used to determine the height of the iceberg while landed on its surface. Satellite imagery was later used to obtain more precise measurements of iceberg dimensions. A compass heading measurement of the orientation of the tracking beacon was also recorded at the time of deployment (Figure 5c), which can be used to determine the orientation of the iceberg and to track changes in its relative rotation over time. When weather conditions permitted, aerial photo surveys of the iceberg were performed to further assist in classifying the morphology of the iceberg and for later identification in satellite imagery.

During the 2019 field campaign deployments, it was possible to revisit an iceberg previously instrumented with ITB 300434063411050 in 2018. After an inspection of the enclosure for any signs of damage or water ingress, it was determined that the system was in good working condition, did not require servicing, and was left to continue its monitoring of the iceberg.

## 3. Results and Discussion

### 3.1. Iceberg Drift

Since their deployment, Cryologger ITBs have achieved up to 1530 days of continuous operation. Collectively, these ITBs have transmitted more than 125,000 GNSS positions and travelled a combined distance of over 16,500 km. The total approximate cumulative drift distances for each beacon are shown in Table 3. Results have shown that the drift patterns and speeds differed considerably between iceberg targets, with a maximum measured drift speed of 6.0 km/h and a median drift speed for all beacons (excluding those deployed on glaciers) of 0.3 km/h.

#### 3.1.1. 2018 Deployments

**Figure 6:**
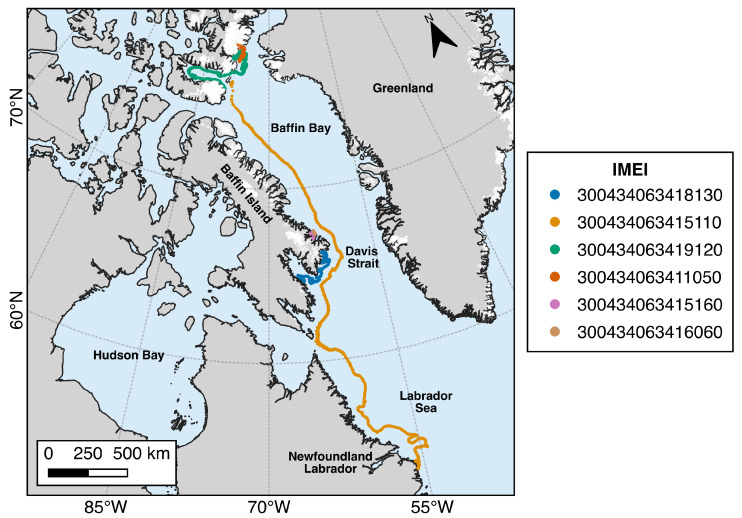
Iceberg drift observations recorded by Cryologger ITB deployments made during the 2018 field season.

Beacon deployments made during the 2018 field season were focused on the areas of Talbot Inlet and waters adjacent to Bylot Island and southern Baffin Island (Figure 3). The recorded iceberg drift tracks are shown in Figure 6. In terms of cumulative distance travelled, the most notable was beacon 300434063415110, which was deployed on a very large tabular iceberg near Bylot Island on 27 August 2018. After remaining grounded for several months, the iceberg became dislodged during the winter of 2019 and travelled over 4000 km southwards before grounding and breaking up off the coast of Labrador in May 2019. Beacon 300434063419120 was deployed on 28 August 2018 on an ice island with a surface area of 5 km^2^ that calved from the Petermann Glacier in 2017 and remained in the general area of northern Baffin Bay until the summer of 2020 when it became trapped in a gyre and eventually broke apart. Satellite imagery confirmed that the beacon remained on a smaller iceberg fragment that continued to drift around Jones Sound before breaking apart on the coast of Devon Island. Beacon 300434063418130 was deployed on an iceberg near southern Baffin Island, which drifted through several fiords before breaking apart in the winter of 2019. Beacons 300434063416060 and 300434063415160, which were deployed on icebergs near Qikiqtarjuaq, Nunavut, were unable to escape the local shallow coastal area and frequently became grounded. Beacon 300434063415160 eventually travelled further into a nearby fiord and washed up onshore after becoming dislodged from the iceberg as it deteriorated. This beacon provided an opportunity to test the durability of the electronics and capacity of the battery pack and continued to operate until the battery was depleted in August 2022. This ITB was set to transmit every 3 h, and from the long times it took to transmit, it was evident the beacon had difficulty transmitting from the shore, which was likely due to snow and sand cover, as well as a reduced sky view from its location in a fiord with steep sidewalls. Despite this, it was still able to achieve 4 years of continuous operation in less-than-ideal conditions.

#### 3.1.2. 2019 Deployments

**Figure 7:**
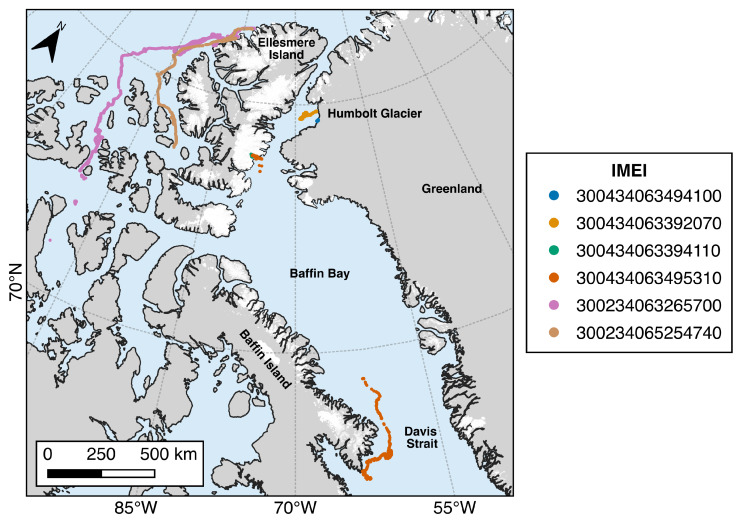
Iceberg drift observations recorded by Cryologger ITB deployments made during the 2019 field season.

Deployments made in 2019 were focused on the regions of Milne Fiord and Talbot Inlet on Ellesmere Island and the Humboldt and Petermann glaciers on the western coast of Greenland (Figure 3). The iceberg tracks reveal that many of the icebergs did not experience significant drift, except those deployed near the northern coast of Ellesmere Island (Figure 7). Beacons 300434063494100 and 300434063392070, deployed on large tabular icebergs near the terminus of the Humboldt Glacier, drifted slowly in looping patterns but remained within 100 km of their initial positions. Beacons 300434063394110 and 300434063495310 were deployed on icebergs near the terminus of the Trinity Glacier in Talbot Inlet. Beacon 300434063394110 drifted slowly across Talbot Inlet until July 2020, when the iceberg catastrophically broke apart, and the beacon was lost. Beacon 300434063495310 was able to drift out of Talbot Inlet but temporarily ceased transmitting in October 2019. When the beacon began transmitting again in May 2020, it had traveled over 1000 km to the southeast to southern Baffin Island. The iceberg continued to drift around the tip of Baffin Island, where it became grounded on a small island and eventually deteriorated. Of the four beacons deployed on Petermann Glacier, three ceased transmitting after the beacons became inverted, likely due to being blown over by a strong wind gust. One remaining deployed beacon, 300434063292950, continues to measure the daily displacement of the glacier (as of December 2023).

### 3.2. Operation

The record of continuous days of operation achieved by the Cryologger ITBs and suspected causes leading to the failure of the beacon is shown in Table 3. Lifespans greatly exceeded expectations, with v1.0 beacons deployed in 2018 ranging from 310 days to 4 years, and v2.0 beacons deployed in 2019 ranging from 333 days to 4 years 70 days, with one beacon still active as of December 2023. The most frequent causes of loss of communication with the ITBs were the eventual deterioration (calving or rolling) or catastrophic breakup of the iceberg and subsequent destruction of the beacon. In many cases, it was possible to use remote sensing imagery to observe major breakup events of icebergs that immediately resulted in transmissions from the beacon ceasing.

**Figure 8:**
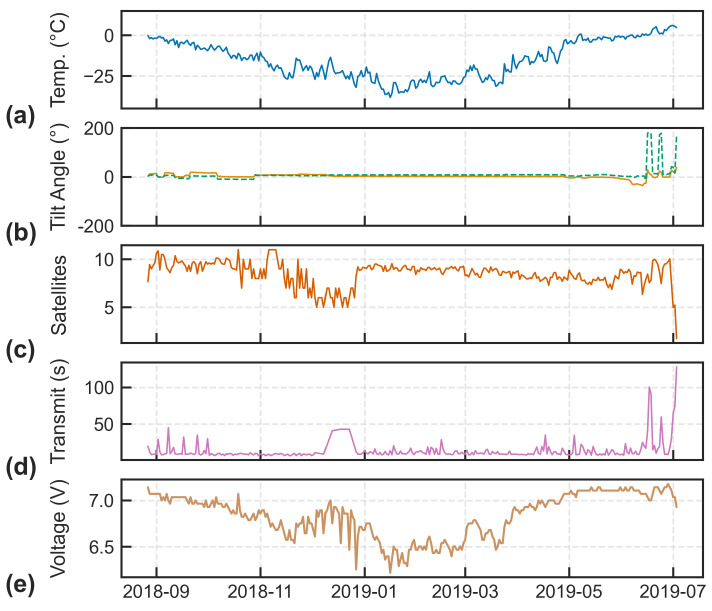
Diagnostic variables recorded from ITB 300434063415110 between 27 August 2018 and 3 July 2019 showing: (**a**) daily mean internal temperature; (**b**) daily maximum pitch (solid orange) and roll (dashed green) angles; (**c**) daily mean number of GPS satellites in view; (**d**) daily mean transmission durations; (**e**) daily minimum battery voltages.

Data collected from the onboard sensors provided valuable information to assess the overall operation of the beacons and determine the environmental conditions to which they were exposed. Figure 8 displays diagnostic variables recorded from ITB 300434063415110, deployed in 2018, which are considered typical. Sensors monitoring the interior conditions of the enclosures (not necessarily representative of the ambient air temperature) revealed that temperatures regularly dropped below −25 °C during the winter months and reached as low as −40 °C (Figure 8a). Under these conditions, the minimum battery voltages were also recorded (Figure 8e), decreasing as low as 6.0 V, but once temperatures increased, voltages quickly returned to nominal (7.2 V). The GNSS receivers were consistently able to track 8 or more satellites and quickly acquired position fixes, impressive considering they only support the Global Positioning System (GPS) constellation; however, during the winter months, fewer satellites were observed (Figure 8c) likely due to the presence of snow cover. Satellite transmissions were very efficient, thanks to the high number of Iridium satellites converging at the North Pole, with most messages transmitting in less than 10 s (Figure 8d). However, issues such as snow cover or a sub-optimal orientation of the beacon enclosure could drastically increase transmission times. The orientation of the beacon was confirmed using the accelerometer pitch and roll angles, with Figure 8b showing three separate instances of roll angles of ±180°, indicating the beacon had become inverted. The orientation, together with the delayed transmission times (i.e., >180 s), became good indicators of when the iceberg may have deteriorated and/or the beacon had become dislodged from the iceberg’s surface.

**Figure 9:**
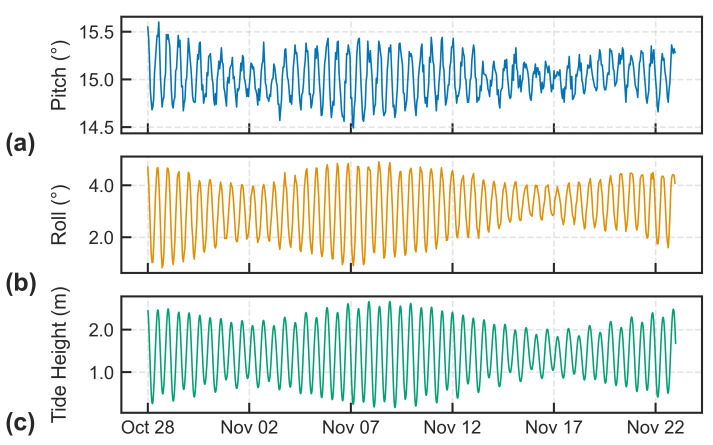
Accelerometer measurements recorded between 27 October to 27 November 2018 from Cryologger ITB 300434063418130 on a grounded iceberg showing (**a**) pitch angle; (**b**) roll angle; (**c**) Tidal height predictions from Cape Dyer, Nunavut, approximately 150 km away.

An interesting discovery from the data recorded from beacon 300434063418130, situated on a grounded iceberg in a fiord along the southern coast of Baffin Island, revealed that the accelerometer detected a repeating signal that varied in both frequency and magnitude. When the daily pitch (Figure 9a) and roll (Figure 9b) measurements were examined over a month and compared with predicted tidal heights (Figure 9c) at Cape Dyer, Nunavut, approximately 150 km from the beacon’s position, it was found to be in phase with the diurnal and lunar-phase components of the tidal cycle. That the accelerometer was sensitive enough to detect variations in the pitch and roll of only 1–2° is a testament to the suitability of new low-cost sensors for measuring the orientation of icebergs and studying the movement of icebergs within three-dimensional space.

Although the majority of ITBs experienced nominal operation throughout their lifespans, a small number of issues were discovered in both versions. In v1.0, erroneous temperature and pressure measurements were recorded by four beacons, sporadically affecting approximately 0.5% of observations. The cause of this error is unknown but is likely due to an intermittent I^2^C sensor bus communication failure. The most significant issue was in v2.0, when a loss of communication with all eight beacons was experienced in November 2019. The cause of this issue was initially suspected to be related to the onset of winter and the inability of the RockBLOCK transceiver’s integrated patch antenna to transmit through a snow cover. However, once the beacons began transmitting again in April 2020, it was determined that the issue was related to temperature and power. In v2.0, the RockBLOCK was powered by a single 3.3 V 600 mA step-down voltage regulator, which, at temperatures below −20 °C, could no longer provide enough power to successfully power up the RockBLOCK to transmit data. Despite 3.3 V being listed as a supported voltage, after consulting the manufacturer, Ground Control, it was determined that the RockBLOCK requires a minimum of 5 V to be supplied when operating in environments with low temperatures. This behaviour was consistent over time, with loss of communications occurring again in the fall of 2021 and 2022. To address this issue, all future Cryologger versions include a 5 V voltage regulator.

An additional issue encountered with a limited number of v2.0 beacons was a tendency to become inverted after deployment, which led to the eventual ingress of water in shallow meltwater ponds and subsequent failure of the electronics. Measurements from the accelerometer were used to confirm when the beacons had flipped upside down. In this orientation, it was observed that the recorded GNSS positions were less accurate, and Iridium transmissions also took longer and failed more frequently. A decrease in temperature inside the enclosure indicated that water ingress occurred before the loss of communication. This was likely a result of the enclosure used in v2.0, which was smaller and lighter and subsequently more susceptible to being blown over by strong wind gusts than the larger and heavier enclosure used in v1.0. Having learned from this, we now recommend attaching the case to a wooden cross or similar structure.

## 4. Conclusions

We have presented the design and some sample results from our low-cost, open-source iceberg drift tracking beacon constructed using off-the-shelf components. The overall success of the Cryologger Ice Tracking Beacon deployments has demonstrated that low-cost, open-source hardware and software can provide a robust platform for the collection of in situ iceberg tracking data. With a total cost of under 650 USD in materials each, this represents a cost-effective alternative to comparable proprietary commercial systems that can track icebergs and relay ancillary in situ data, such as temperature and tilt.

The development and deployment of these novel iceberg tracking beacons was intended to enhance and supplement existing iceberg observation networks within the Canadian Arctic. Data collected from Cryologger ITBs has already contributed to a database of iceberg tracking beacon tracks compiled by Carleton University and the Canadian Ice Service [40]. It has also provided key insights regarding iceberg drift characteristics and has improved our understanding of iceberg distributions within Canada’s navigable waters. Most importantly, this research demonstrates to the scientific community that inexpensive, open-source hardware and software can provide a viable solution for the long-term monitoring of icebergs and ice island drift patterns in the polar regions. We hope that the technology developed by this project will lower the cost of research, encourage the adoption of open-source technologies for scientific research, and enable innovation in the polar science community.

Development of the newest version of the Cryologger ITB is currently underway and is focused on improving overall reliability, modularity, and ease of construction. As lithium batteries are challenging to transport and more environmentally harmful than other battery chemistries, we are exploring the use of more environmentally friendly materials. We are also examining improvements to waterproofing that will allow the tracking beacons to transform into ocean-drifter buoys once the breakup of the icebergs occurs. New research applications are also constantly being explored for the Cryologger platform, which has already been configured for use as an automatic weather station to provide weather services to northern communities.

## Figures and Tables

**Table 1:** Cryologger ITB electronics components and estimated costs current as of December 2023. Taxes and shipping are not included. Total costs for each version are shown in bold.

Component	Product	Version	Cost (USD)
Satellite transceiver	RockBLOCK 9603N	1.0, 2.0	$272
Satellite transceiver antenna	Maxtena M1621HCT-P-SMA	1.0	$54
Microcontroller	Adafruit Pro Trinket 3V 12 MHz	1.0	$10
	Adafruit Feather M0 Basic Proto	2.0	$20
GNSS receiver	Adafruit Ultimate GPS Breakout	1.0	$30
	Adafruit Ultimate GPS FeatherWing	2.0	$25
Accelerometer/magnetometer	Adafruit LSM303	1.0	$15
	Pololu LSM303D	2.0	$8
Temperature/pressure sensor	Adafruit MPL3115A2	1.0	$10
Real-time clock	Maxim DS3231SN + Adafruit SMT Breakout	1.0	$16
	Adafruit DS3231 Precision RTC FeatherWing	2.0	$14
Voltage regulator	Pololu 5 V, 2.5 A D24V22F5	1.0	$12
	Pololu 3.3 V, 600 mA D36V6F3	2.0	$7
Enclosure	Nanuk 905	1.0	$74
	Nanuk 903	2.0	$48
Battery	Tadiran TLP-93121-B-AL1	1.0, 2.0	$145
**Totals**		**1.0**	**$638**
		**2.0**	**$539**

**Table 2:** Cryologger ITB recorded variables transmitted via Iridium Short Burst Data messages.

Variable	Unit	Description	Size (Bytes)
unixtime	s	Unix time (seconds since 1970-01-01 epoch)	4
temperature_int	°C	Internal temperature	2
pressure_int	hPa	Internal pressure	2
pitch	°	Pitch angle	2
roll	°	Roll angle	2
heading	°	Tilt-compensated magnetic heading (0–360°)	2
latitude	°	GNSS latitude	4
longitude	°	GNSS longitude	4
satellites		Number of GNSS satellites in view	2
hdop		GNSS horizontal dilution of precision	2
voltage	V	Battery voltage	2
transmit_duration	s	Transmission time of SBD message	2
message_counter		Number of transmitted messages	2

**Table 3:** Record of continuous days of operation, cumulative distance travelled, and causes of loss of communication of Cryologger ITB deployments made during the 2018 and 2019 field seasons. Current as of December 1, 2023.

IMEI	Deployment Date (yyyy-mm-dd)	Hardware Revision	Days Operational	Positions Recorded	Distance Travelled (km)	Cause of Termination
300434063418130	2018-09-03	1.0	435	10,231	506.2	Iceberg deterioration
300434063415110	2018-08-27	1.0	310	5670	4037.1	Iceberg deterioration
300434063419120	2018-08-28	1.0	709	10,665	2043.3	Iceberg deterioration
300434063411050	2018-08-28	1.0	748	10,491	616.7	Iceberg deterioration
300434063415160	2018-09-01	1.0	1460	28,944	417.3	Battery depletion
300434063416060	2018-09-01	1.0	386	1915	90.7	Iceberg deterioration
300234063265700	2019-08-01	2.0	1170	16,700	3352.3	Iceberg deterioration
300234065254740	2019-08-01	2.0	738	14,903	2270.7	Inversion/water ingress
300434063496100 *	2019-08-01	2.0	1401	1272	4.9	Inversion/water ingress
300434063392350 *	2019-08-01	2.0	399	621	1.4	Inversion/water ingress
300434063292950 *	2019-07-30	2.0	1530	1566	5.5	Currently active
300434063498160 *	2019-07-30	2.0	1402	1684	4.9	Inversion/water ingress
300434063494100	2019-08-05	2.0	803	5236	234.3	Inversion/water ingress
300434063392070	2019-08-05	2.0	1503	9963	529.7	Iceberg deterioration
300434063394110	2019-08-01	2.0	331	5220	169.5	Iceberg deterioration
300434063495310	2019-08-01	2.0	431	3272	2534.9	Iceberg deterioration

* Beacon deployed on Petermann Glacier, Greenland.

## Data Availability

Plans for the construction of the Cryologger ITB are available at https://github.com/cryologger/ice-tracking-beacon. Data will be made available in the Iceberg Tracking Beacon Database at the Polar Data Catalogue.
